# Kombucha: Formulation, chemical composition, and therapeutic potentialities.

**DOI:** 10.1016/j.crfs.2022.01.023

**Published:** 2022-02-04

**Authors:** Jayme César da Silva Júnior, Ísis Meireles Mafaldo, Isabelle de Lima Brito, Angela Maria Tribuzy de Magalhães Cordeiro

**Affiliations:** a Department of Food Technology, Federal University of Paraíba, João Pessoa, PB, Brazil; b Department of Agroindustrial Management and Technology, Federal University of Paraíba, João Pessoa, PB, Brazil

**Keywords:** Fermented tea, Bioactive compounds, Fermented beverage, Analog kombucha

## Abstract

Kombucha is a millennial beverage with great potential due to its functional claims. The infusion of black or green tea leaves (*Camellia sinensis*) and sugar is fermented by a symbiotic culture of bacteria and yeasts (SCOBY) resulting in an acidic and lightly carbonated beverage, kombucha. It offers in its composition phytoconstituents with relevant nutritional valor, among these, flavonoids that stand out for their antioxidant, anti-inflammatory characteristics and their association with decreasing the risks of various diseases. Previous studies *in vivo* and *in vitro* have shown promising results using kombucha as a functional beverage. Those studies promote the search for alternative raw materials for the production of kombucha, in addition, new ingredients interfere in the production, constitution, and nutritional potentialities of the beverage, as well as its functionality in the face of diseases. Thus, this graphical review compiles relevant scientific data on kombucha involving its origin, production, nutritional potential, and possible health benefits associated with its consumption.

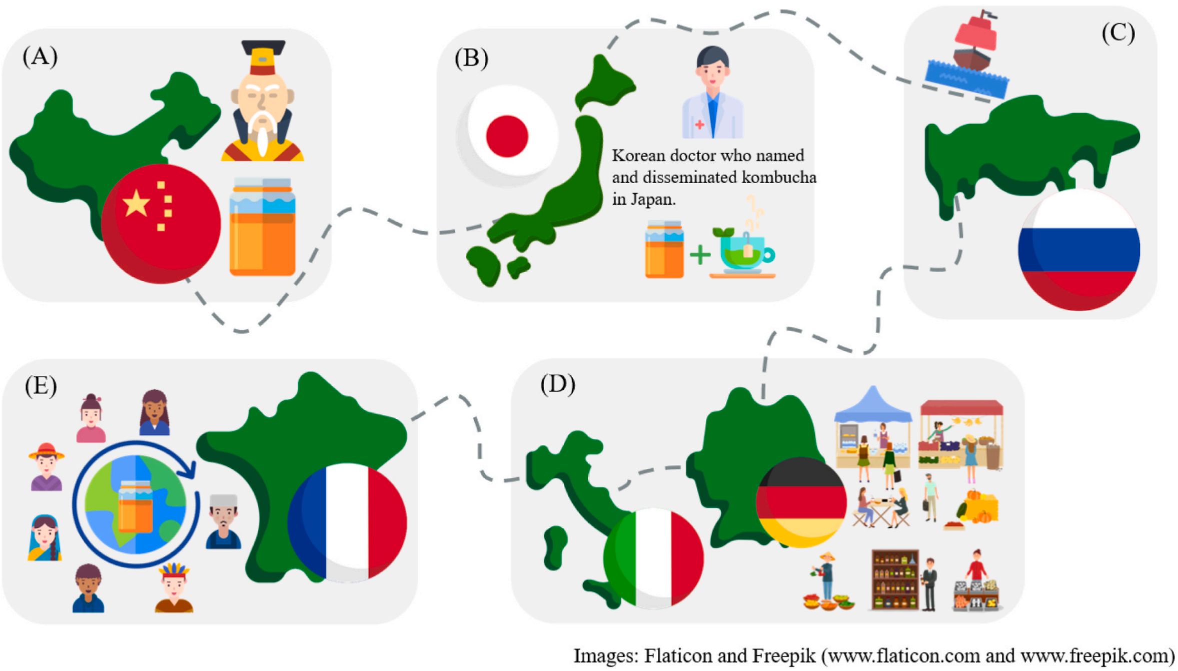

**Fig. 1. The historical context of kombucha, its origin, and expansion.** Records show that kombucha has been a product appreciated for thousands of years, (A) and its consumption began around 220 BC., in Manchuria, located in northeastern China. During the Chinese dynasty "*Tsin*", the beverage became popular in the country, as it was already believed in its energy and detoxifying properties. (B) The Korean doctor named Kombu, who used the beverage to treat intestinal problems of the emperor at the time, around the year 414 AD. introduced the beverage in Japan. After this, the beverage gained prominence and came to be called "Kombucha" in honor of the doctor Kombu (Chakravorty et al., 2019a,b). (C) Given its nutritional and functional potentialities, kombucha spread throughout the world and became also known in Europe. Studies report that it first arrived in Russia via commercial sea routes and (D) expanded to Germany and Italy in the 20th century, shortly after World War II. In the 1950s, (E) Kombucha also became popular in France and North Africa (Jayabalan et al., 2016). Currently, kombucha has gained prominence again and has been widely disseminated in the world market for beverages and products with functional claims (Dutta and Paul, 2019).

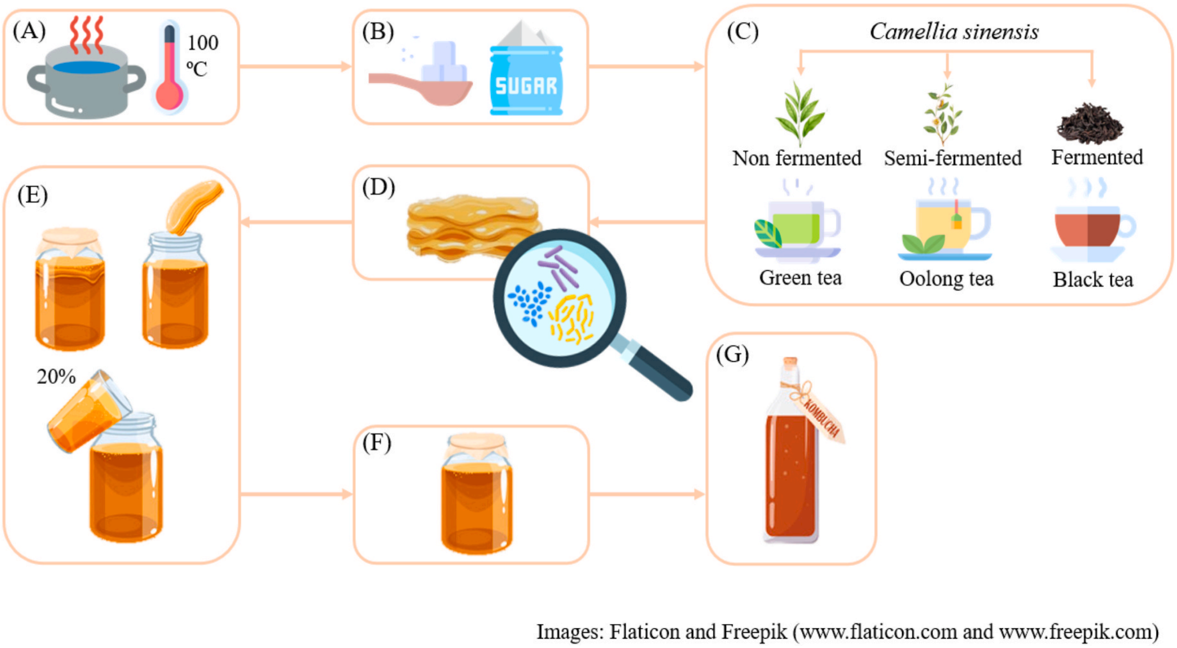

**Fig. 2. Formulation of traditional kombucha.** The process of obtaining kombucha is considered simple since it does not require large equipment and hard-to-reach ingredients. According to the region and the studies developed, there are variations in the specificities and proportions of the materials used, but the methodology described by Jayabalan et al. (2014) is referred to as the standard process (Dutta and Paul, 2019). The elaboration process of kombucha starts from the preparation of the tea, by infusion of *Camellia sinensis* leaves (green tea, black tea, or oolong tea), then the sugar is added and, at room temperature, a cellulosic film called "Symbiotic Culture of Bacteria and Yeasts" (SCOBY) is inoculated. In this film there is a predominance of acetic acid bacteria and yeasts, the SCOBY is responsible for fermentation and gives the characteristics of the beverage. Some authors also report the use of previously fermented tea as starters, which can be used to start the fermentation process (Chakravorty et al., 2019a,b; Jayabalan et al., 2014, 2016; Kapp and Summer, 2019).

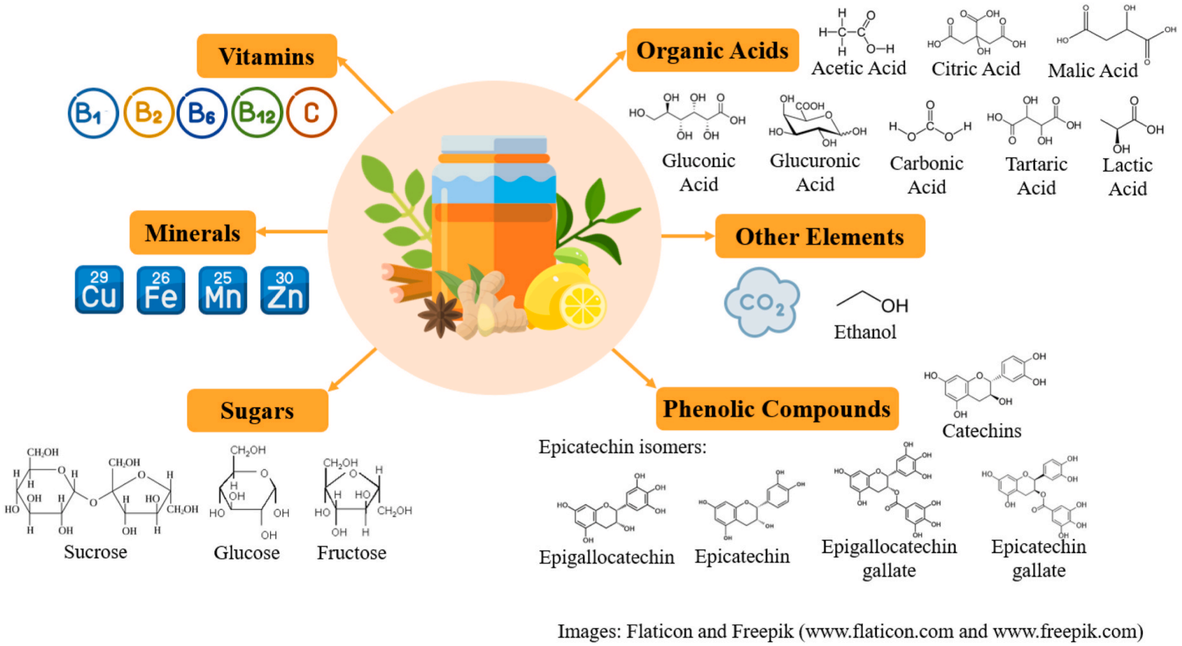

**Fig. 3. Chemical composition of traditional kombucha.** Kombucha has interesting nutritional valor, mainly due to the benefits of *Camellia sinensis* that are already well described in many literatures. As for the characteristic acidity of the beverage, it may vary according to the time and speed of fermentation and occurs due to the production of organic acids, especially acetic acid (Chakravorty et al., 2016; Vitas et al., 2018). Acetic bacteria, the major part of the SCOBY, synthesize acetic acid from the ethanol produced by yeasts. However, there are other acids in kombucha, such as citric, malic, gluconic, glucuronic, carbonic, tartaric, and lactic, among others (Chakravorty et al., 2019a,b; Vitas et al., 2018; Zubaidah et al., 2019). Others compounds are found in kombucha; ethanol, sugars, mainly glucose and fructose, but also sucrose fractions that are not degraded by yeasts. As well as amino acids, vitamins of the B and C complex, minerals such as iron, zinc, and manganese, and polyphenols that may vary according to the ingredients used and the conditions of the fermentation (Abuduaibifu and Tamer, 2019; Jayabalan et al., 2014, 2016; Rahmani et al., 2019; Villarreal-Soto et al., 2018). Therefore, studies show that the chemical composition of the beverage is directly linked to the ingredients and their proportions, as well as the variation of the fermentation parameters. Thus, these variations can potentiate the production of specific nutritional compounds. Examples include phenolic compounds, known to have several health benefits and are associated with disease prevention (Aspiyanto et al., 2017). Among the compounds found in kombucha are flavonoids, especially catechins and their derivatives, considered functional substances (Chakravorty et al., 2019a,b; Jayabalan et al., 2016; Kapp and Sumner, 2019; Leal et al., 2018).

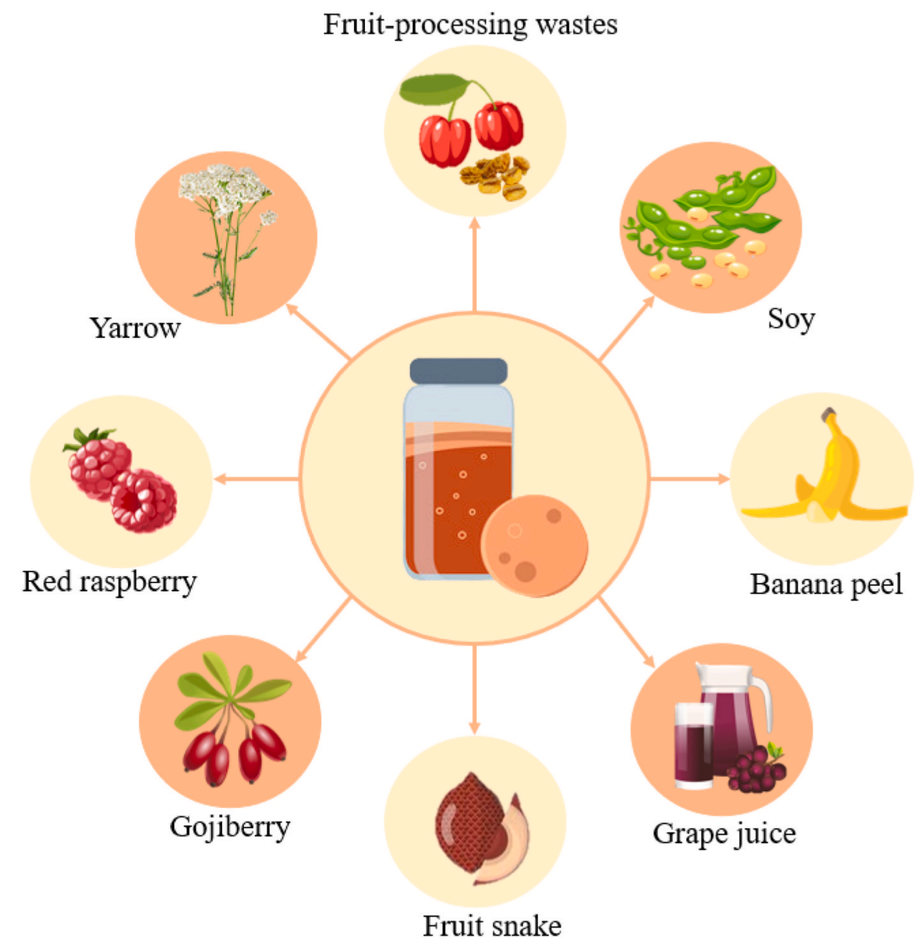

**Fig. 4. Use of alternative substrates in the preparation of analogous kombucha.** Because of the popularization and visibility of kombucha, researchers have been investigating the variation not only of the concentrations of the ingredients of its original formulation but of new raw materials and processes. These innovations increase the possibility of flavors and functionalities of the beverage, which further contributes to the acceptance of these products, called kombucha analogs. Given this reality, current research has been replacing *Camellia sinensis* or associating it with other herbs, fruits, and vegetables for the production of the beverage. Those tests have occurred, either using these raw materials to prepare the direct infusion and/or its addition to induce a second fermentation, which favors the flavoring and acceptance of the final product, besides potentiating the profile of bioactive compounds of the beverage (Emiljanowicz and Malinowska-Pańczyk, 2019). The use of soy (Xia et al., 2019), yarrow (Vitas et al., 2018), processed fruit residues (Leonarski et al., 2021), red raspberry (Ulusoy and Tamer, 2019), banana's peel (Pure and Pure, 2016), grape juice (Ayed et al., 2017), goji berry (Abuduaibifu and Tamer, 2019), snake fruits (Zubaidah et al., 2018) and, more recently, the use of umbu-cajá and pitanga pulp (da Silva Júnior et al., 2021) have been reported in the literature.

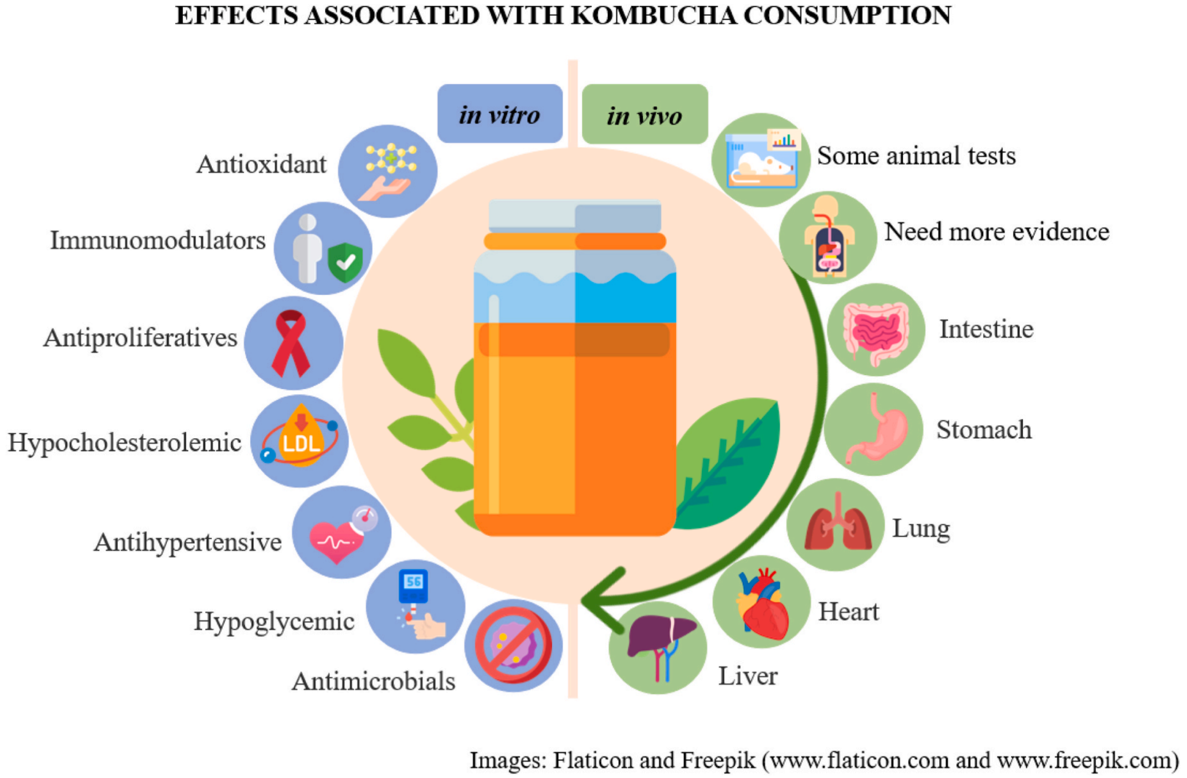

**Fig. 5. Effects associated with kombucha consumption.** The biological activities of kombucha and their respective functional and therapeutic potential have been associated with the beverage's chemical constituents and are commonly reported in studies involving *in vitro* and/or *in vivo* tests. Numerous *in vitro* tests have already been reported and found that the kombucha beverage has mainly antioxidant activity, which is well documented in the literature and is mainly associated with the plant used, being endorsed by various methods. Other biological activities linked to the consumption of kombucha have been reported and documented, such as immunomodulatory, antihypertensive, hypocholesterolemic, hypoglycemic, antiproliferative, and antimicrobial. Some tests were developed *in vivo* using animals. However, currently, there are no large studies involving direct effects on the human body associated with the ingestion of kombucha, both in the general shape of the organs and in a specific organ, making it a major obstacle in scientific evidence. However, there are records in the literature about the effect of kombucha on human cells (*in vitro*) (Kapp and Sumner, 2019; Morales, 2020; Sinir et al., 2019).

## Acknowledgments

The authors would like to thank CNPq-Brazil (Process Number 132839/2019–9) and CAPES-Brazil (Finance Code 001) for the scholarships granted to authors who are part of the Graduate Program in Food Science and Technology - Federal University of Paraíba (PPGCTA - UFPB).

## CRediT authorship contribution statement

**Jayme César da Silva Júnior:** Conceptualization, Methodology, Investigation, Data curation, Writing – original draft, Visualization, Image editing. **Ísis Meireles Mafaldo:** Conceptualization, Data curation, Writing – original draft, Image editing; Final image. **Isabelle de Lima Brito:** Conceptualization, Methodology, Resources, Writing – original draft, Writing – review & editing, Supervision, Project administration, Funding acquisition. **Angela Maria Tribuzy de Magalhães Cordeiro:** Conceptualization, Methodology, Resources, Writing – original draft, Writing – review & editing, Supervision, Funding acquisition.

## Declaration of competing interest

The authors declare that they have no known competing financial interests or personal relationships that could have appeared to influence the work reported in this paper.
